# Molecular modeling of cornulin (CRNN) for docking with phytocompounds from Justicia adhatoda L.

**DOI:** 10.6026/97320630017200

**Published:** 2021-01-31

**Authors:** Jayaraman Selvaraj, Shazia Fathima JH, Venkatacalam Sivabalan, Umapathy Vidhya Rekha, Rajagopal Ponnulakshmi, Veeraraghavan Vishnupriya, Malathi Kullappan, Radhika Nalinakumari Sreekandan, Surapaneni Krishna Mohan, Periyasamy Vijayalakshmi

**Affiliations:** 1 Department of Biochemistry, Saveetha Dental College and Hospitals, Saveetha Institute of Medical and Technical Sciences, Saveetha University, Chennai - 600 077, India; 2 Department of Oral and Maxillofacial Pathology, Ragas Dental College and Hospitals, Chennai, India; 3 Department of Biochemistry, KSR Institute of Dental Sciences and Research, Thiruchengodu-637215, India; 4 Department of Public Health Dentistry, Sree Balaji Dental College and Hospital, Pallikaranai, Chennai-600 100, India; 5 Central Research Laboratory, Meenakshi Academy of Higher Education and Research (Deemed to be University), Chennai-600 078, India;; 6 Department of Research, Panimalar Medical College Hospital & Research Institute, Varadharajapuram, Poonamallee, Chennai - 600 123, India; 7 Department of Clinical Skills & Simulation, Panimalar Medical College Hospital & Research Institute, Varadharajapuram, Poonamallee, Chennai - 600 123, India; 8 Department of Biochemistry and Department of Clinical Skills & Simulation, Department of Research, Panimalar Medical College Hospital & Research Institute, Varadharajapuram, Poonamallee, Chennai - 600 123

**Keywords:** Oral Squamous Cell Carcinoma, cornulin, molecular modeling, molecular docking

## Abstract

Cornulin (CRNN) is linked with tumour progression. Therefore, it is of interest to document data on the molecular modeling of cornulin (CRNN) for docking with phytocompounds (Pyrazinamide, Anisotine, Vasicinone, Vasicoline) from Justicia adhatoda L. Thus, we document the optimal binding features of these compounds with the cornulin model for further consideration.

## Background:

Cornulin (CRNN) is linked with tumour progression [1-7]. Therefore, it is of interest to document data on the molecular modeling of cornulin (CRNN) for docking with phytocompounds (Pyrazinamide, Anisotine, Vasicinone, Vasicoline) from Justicia adhatoda L.

## Materials and Methods:

### Sequence retrieval and 3D model building:

The full amino acid (495aa) sequence of CRNN is downloaded from the Uniprot Knowledgebase database in FASTA format with accession number Q9UBG3. The NCBI Simple Local Alignment Search Tool (Psi-BLAST) [8] was used to search the Protein Databank (PDB) for templates. The template with PDB ID: 4PCW was chosen having a 41.76 percent identity score. The Swiss model server was used for creating the protein model.

### Model evaluation:

ProCheck [9] was used for model validataion.

### Prominent binding site prediction:

The Cavity Plus server [10] was used to identify the binding pockets.

### Ligand retrieval:

The structure data for 12 compounds from Justicia adhatoda L was downloaded from PubChem database. All the compounds were downloaded in SDF format and converted to the PDB format using Pymol.

### Molecular docking and interaction analysis:

Molecular docking and visualization were done using a standard procedure using PyRx, AutoDock 4 and Pymol [11-13].

## Results and Discussion:

**Figure 1:**
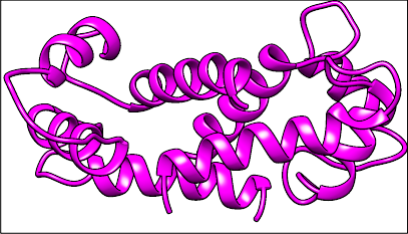
Structure of cornulin model.

**Figure 2:**
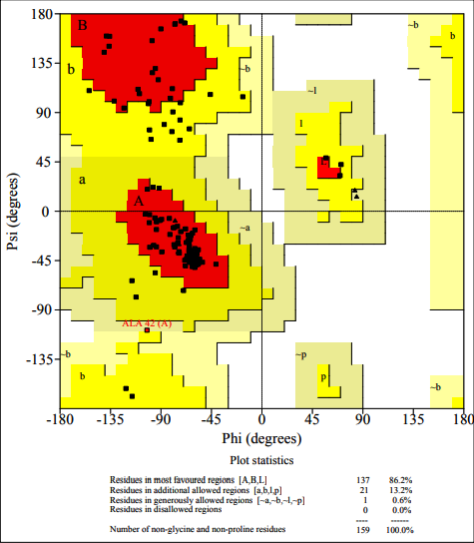
Ramachandran plot analysis of cornulin model.

**Figure 3:**
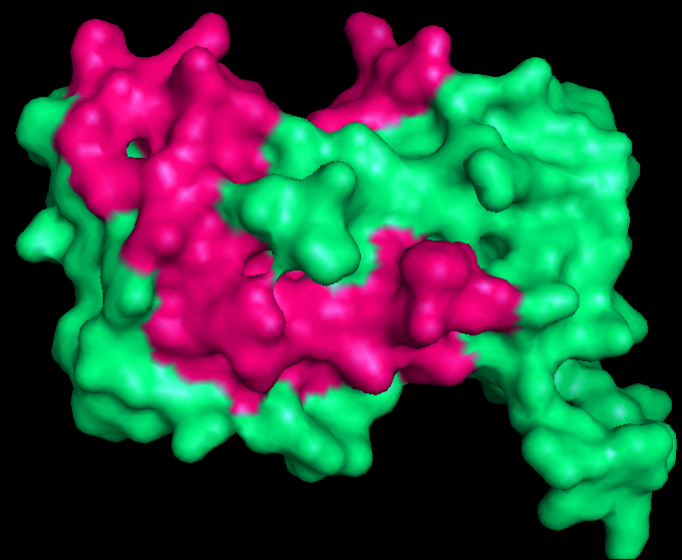
Predicted active site region (Pink color represent the predicted binding site region).

**Figure 4:**
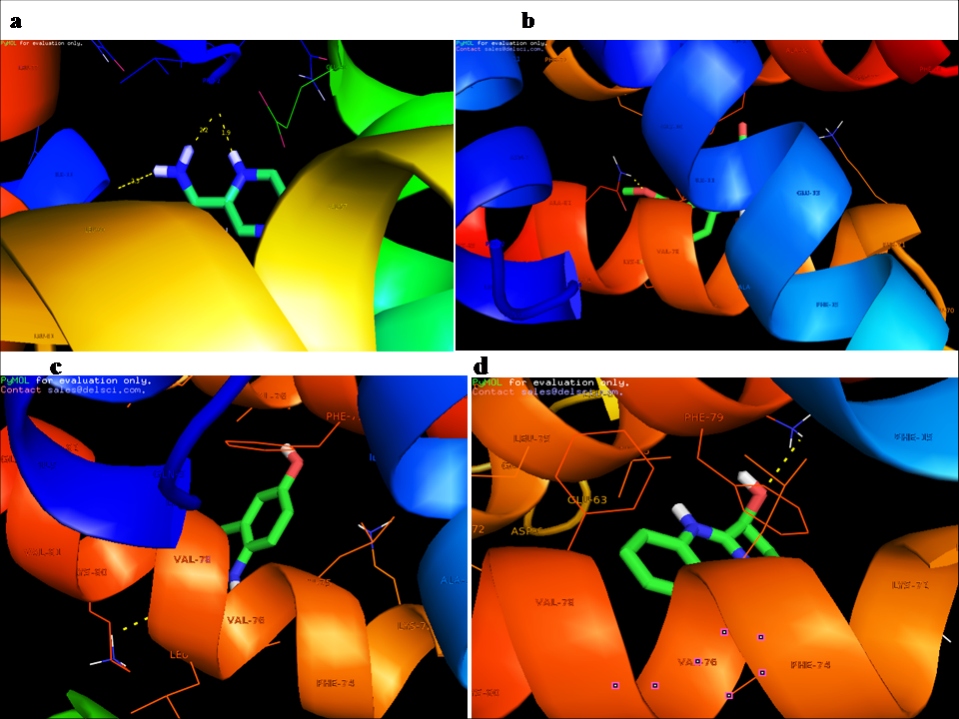
Molecular interaction of cornulin with (a) Pyrazinamide; (b) Anisotine; (c) Vasicinone and (d) Vasicoline.

The SWISS-MODEL homology \cornulin was created (Figure 1) using a known structure (PDB ID 4PCW) with 41.7% sequence identity as a template evaluated using Ramachandran plot analysis (Figure 2). Cavity Plus was used to predict binding pockets (Figure 3). The binding pockets are made of PRO: 2, GLN: 3 LEU:4, LEU:5: GLN:6: ILE:8: ASN:9: GLY:10: ILE:11, ILE:12, GLU:13, ALA:14, ARG:16, LEU:37, GLU:38, GLN:39, GLU:40, PHE:41, ALA:42, ASP:43, VAL:44, ILE:45, LEU:77, LYS:80, VAL:81, ALA:82, ALA:84, CYS:85, PHE:86, LYS:87, THR:88 and LEU:89.. Molecular docking of the protein model with the compounds shows that PRO-2, GLY-10, GLN-83, LYS-80, LYS-80 residues show strong binding interactions with the phytochemicals (Figure 4) for further consideration in the development of optimal drugs against oral cancer.

## Conclusion:

We document the optimal binding features of phytocompounds (Pyrazinamide, Anisotine, Vasicinone, Vasicoline) from Justicia adhatoda L with Cornulin in the context of cancer for further consideration.
